# Women’s experiences of monitoring the small-for-gestational age fetus by ultrasound: A qualitative study

**DOI:** 10.1371/journal.pone.0216052

**Published:** 2019-05-01

**Authors:** Charlotte A. Vollgraff Heidweiller-Schreurs, Marjon A. de Boer, Karuna R. M. van der Meij, Caroline J. Bax, Christianne J. M. de Groot, Lidewij Henneman

**Affiliations:** 1 Department of Obstetrics and Gynaecology, Amsterdam UMC, Vrije Universiteit Amsterdam, Amsterdam, The Netherlands; 2 Amsterdam Reproduction and Development Institute, Amsterdam UMC, Amsterdam, The Netherlands; 3 Department of Clinical Genetics, Amsterdam UMC, Vrije Universiteit Amsterdam, Amsterdam, The Netherlands; 4 Department of Obstetrics and Gynaecology, Amsterdam UMC, University of Amsterdam, Amsterdam, The Netherlands; Ruprecht Karls University Heidelberg, GERMANY

## Abstract

**Objective:**

To explore experiences among pregnant women diagnosed with a small-for-gestational age (SGA) fetus, and monitored by frequent ultrasounds.

**Methods:**

We performed a qualitative study at the outpatient clinic of the Gynecology and Obstetrics department of a large academic hospital in Amsterdam. Semi-structured interviews were conducted with fifteen women, diagnosed with an SGA fetus during their pregnancy and having had at least two monitoring ultrasounds since. Themes were identified following analysis of the interview transcripts.

**Results:**

Most women experienced the frequent ultrasounds as a source of support providing comfort and a feeling of safety. It was considered necessary, in the best interest of the baby, which outweighed the discomfort caused by having to come to the hospital frequently. Women described anxiety building up prior to each ultrasound, but feeling reassured and relieved afterwards. During the ultrasound a continuous explanation was preferred, which provided confirmation and a feeling of security. Women identified the uncertainty of SGA’s cause and prognosis as one of the biggest challenges to cope with, for which they used different strategies. Many women expressed a need for more detailed information and counselling, including non-medical aspects of pregnancy and delivery as well. Lastly, many women reported that seeing different doctors negatively influenced the perceived quality of care.

**Conclusions:**

In general, women in this study were satisfied with the ultrasounds for their small-for-gestational age pregnancies. However, women expressed a need for additional information to help cope with a feeling of uncertainty regarding cause and prognosis. Their medical team should preferably provide this in a consistent and continuous manner.

## Introduction

A fetus is generally diagnosed as ‘small-for-gestational age’ (SGA) when growing below the 10^th^ percentile at any given gestational age, measured by ultrasound.[1] Consequently, SGA affects approximately 10 percent of all pregnancies, making it a common prenatal problem. SGA is a complex and multifactorial condition.[1] Placental insufficiency is the most common cause in non-anomalous fetuses, and constitutes one of the leading causes of maternal and neonatal morbidity and mortality worldwide.[2–4] However, in the non-anomalous fetus it is difficult to distinguish suboptimal fetal growth due to placental insufficiency from adequate growth of a genetically small fetus. Consequently, the cause and prognosis of SGA are often uncertain. The complexity of SGA is further exacerbated by the fact that no effective treatment exists, except iatrogenic delivery of the fetus to prevent intrauterine death or irreversible organ damage.[5]

In the Netherlands, almost three-quarters of pregnant women deliver their baby under hospital-led care. Women can be under hospital-led care from the beginning of their pregnancy, or they become transferred from midwife-led to hospital-led care during pregnancy or delivery, for instance in case of SGA.[6] Assessment of fetal size is an established part of antenatal care and is generally performed during each visit by manual palpation of the uterine size. In the Netherlands, two types of ultrasounds are standardly offered to each woman: the first-trimester or term ultrasound (around 9–12 weeks of gestation), and the anomaly scan (around 20 weeks of gestation). After suspected SGA is confirmed by ultrasound biometry, the fetal condition is monitored by frequent ultrasounds in the hospital.

As of yet, the optimal ultrasound frequency and content remain unknown, giving rise to varying protocols both within and across countries.[7] Knowing the experiences of patients may help tailoring such protocols to their needs as well. The importance of patient satisfaction with regard to the monitoring protocol is further stressed by a potential link between increased maternal pregnancy-specific stress and deteriorated fetal and neonatal well-being, suggested in a questionnaire study by Levine et al.[8] More in-depth exploration is required to understand women’s experiences and preferences for ultrasounds as well as to identify issues that impact their level of satisfaction. This study therefore aimed to obtain the views of women as regards the ultrasonographic monitoring of their pregnancies complicated by SGA.

## Methods

### Setting

A qualitative study with semi-structured interviews was conducted in the outpatient clinic of the Obstetrics and Gynecology department of the Amsterdam UMC (location VU University Medical Center (VUMC)), the Netherlands. The consolidated criteria for reporting qualitative research (COREQ) checklist was used to appraise the methodological quality of the study and guided the manuscript preparation.[9] Participants were included based on three conditions: (1) a diagnosis with SGA fetus (i.e. estimated fetal weight or abdominal circumference of less than the tenth percentile), (2) a singleton pregnancy, and (3) a minimum of two conducted ultrasounds since the diagnosis. Women were excluded if the fetus was diagnosed with structural and/or chromosomal abnormalities. The hospital’s Medical Ethical Committee approved the study protocol (reference number 2017.002). All participants provided written informed consent before the interview.

**Fig 1:**
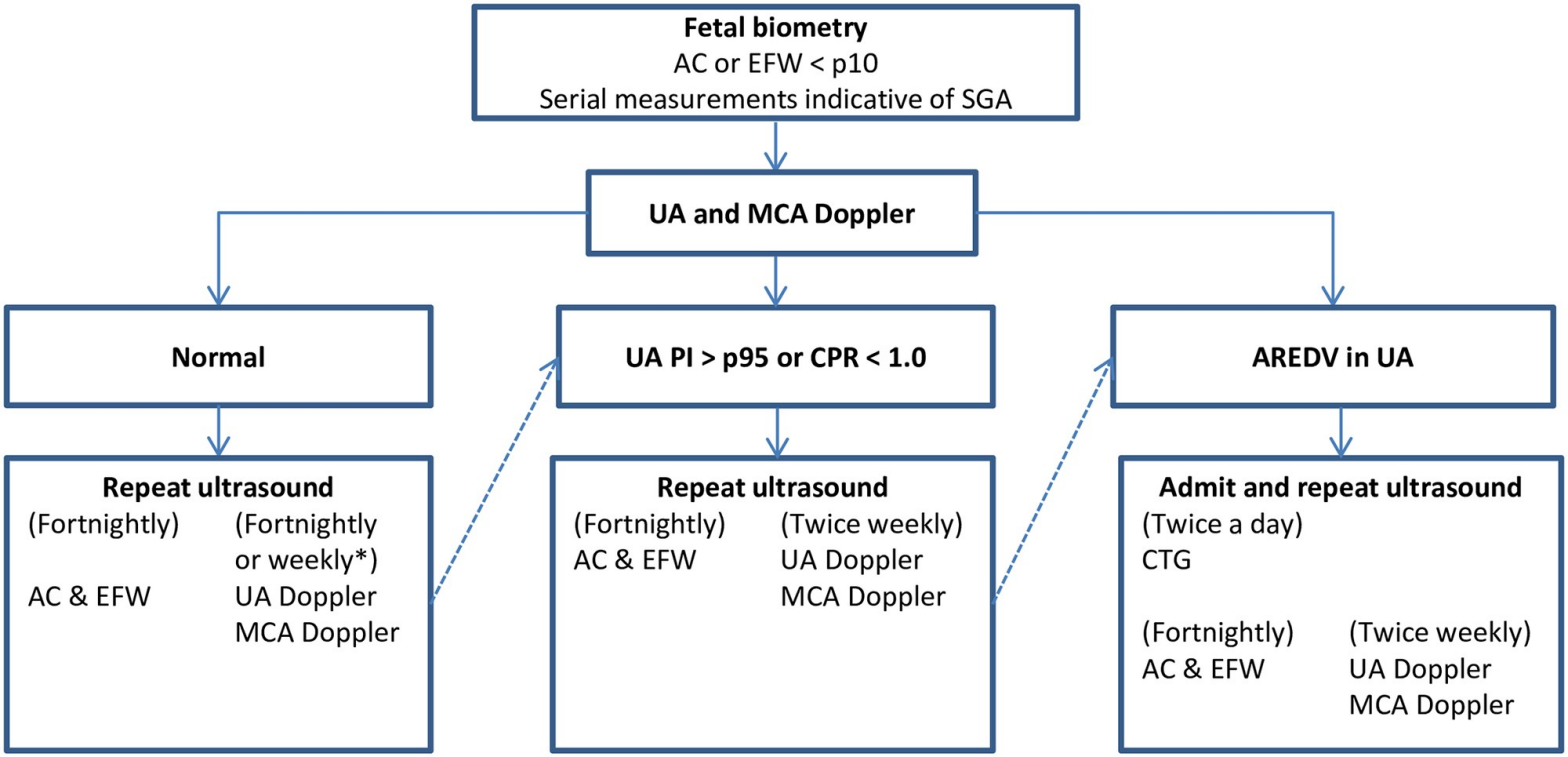
Care pathway for ultrasonographic monitoring of the small-for-gestational age fetus in the Amsterdam UMC, location VUMC, 2017. *Dependent on severity of SGA. Abbreviations: AC, abdominal circumference; EFW, estimated fetal weight; SGA, small-for-gestational age; UA, umbilical artery; MCA, middle cerebral artery; CPR, cerebroplacental ratio; PI, pulsatility index; AREDV, Absent/reversed end–diastolic velocities; CTG, cardiotography.

The local SGA protocol employed in the Amsterdam UMC in 2017 is described in Fig 1 and adheres to international recommendations.[1] Monitoring of SGA took place by serial ultrasound examinations, performed by one of six experienced ultrasonographists. After each ultrasound, a medical doctor (i.e. gynecologist or gynecologist in training) or clinical midwife performed an obstetric consultation. The frequency of ultrasounds ranged from fortnightly to twice weekly, dependent on the severity of the condition, and included measurement of fetal growth, Dopplers of the umbilical artery (UA) and of the middle cerebral artery (MCA), and measurement of the amniotic fluid.

### Participants and recruitment

Patients were recruited by consecutive sampling, and interviews were continued until no new themes or perspectives arose from the interviews. During this period, a total of 45 women were deemed eligible to participate in the study, 37 of which gave oral permission to be contacted by the researcher by email or telephone. Fifteen women chose to participate in the study, after which saturation was reached and further recruitment of participants was stopped. Reasons not to participate were lack of time, emotional burden, not meeting the inclusion criteria and a lack of Dutch and/or English language skills.

Two female researchers (CAVHS and KRMM) conducted the interviews between March and December of 2017. Prior to the interview, the interviewers had not met the participants, and reassured the participants that they were not involved in their medical team and that confidentiality was assured. Interviews lasted between 30 and 60 minutes and followed a semi-structured interview guide consisting of open-ended questions. After the interview, the participants were asked to fill in a short questionnaire assessing several descriptive characteristics (maternal age, gestational age, previous pregnancies/parity, educational level, and country of birth of the participant and the participant’s parents, which were used to identify country of origin[10]). The full interview guide and questionnaire can be found in S1 Appendix. Probing techniques were used to encourage more specific and elaborate responses from the participants.[11]

Characteristics of the 15 interviewed women are shown in Table 1. Maternal age ranged from 26 to 42 years. Education was high in the majority of participants (73%). Fourteen participants were pregnant with a gestational age ranging from 23 to 34 weeks, one interview was done postnatal. Participants could choose the location of the interview. Seven of the interviews took place at the outpatient clinic, four at the participant’s home, and four interviews were conducted by phone. Three of the 15 participants brought another person to the interview. One woman’s partner was present and actively took part in the interview; in the other interviews the accompanying person (one mother-in-law, and one partner during only the first minutes) did not add much.

**Table 1:** Characteristics of participants at the time of interview, n = 15.

Interview #	Age (years)	Gestational age (weeks)	Parity	Education level*	Country of origin	Ultrasound frequency
**1**	37	31	1	High	Colombian	Weekly
**2**	35	34	1	High	Dutch	Weekly
**3**	42	27	0	High	Dutch	Twice weekly
**4**	26	35	0	Medium	Dutch	Weekly
**5**	29	26	0	High	Indian	Fortnightly
**6**	36	25	1	High	Dutch	Weekly
**7**	26	27	0	High	Sri Lankan	Fortnightly
**8**	30	23	0	Medium	Dutch	Weekly
**9**	41	26	1	High	Dutch	Fortnightly
**10**	37	30	2	Medium	Dutch	Fortnightly
**11**	31	postnatal	2	High	Indian	Weekly
**12**	31	32	0	High	British	Twice weekly
**13**	31	30	0	High	Dutch	Twice weekly
**14**	32	29	1	High	Dutch	Weekly
**15**	26	35	0	Medium	Moroccan	Weekly

*Education was defined according to the Central Bureau for Statistics Netherlands[10]: Low: elementary school, lower level of secondary school, lower vocational training; Medium: higher level of secondary school, intermediate vocational training; and High: higher vocational training, university.

### Data preparation and analysis

Audio recordings of the interviews were transcribed verbatim and anonymised. The transcripts of the first two interviews were discussed with four researchers (CVHS, KM, LH and MB), to discuss the first emerging themes and patterns for analysis. The coding was done independently by two researchers (CVHS and KM) for rigour. The analysis started with the identification of initial concepts from the data, which were further classified and clustered into sub-themes and overarching key themes through an iterative process of coding. The software program ATLAS.ti, version 7.5.16, was used for data management and thematic coding. Representative quotes from the interviews are presented to illustrate the findings.

## Results

From the interviews, four major themes were identified and are discussed below: (1) experience of the ultrasound, (2) uncertainty and how to cope with it, (3) information need and support, and (4) continuity of care and satisfaction.

All women but one were under care with a primary midwifery practice prior to the diagnosis of SGA. Discovering something was wrong was generally perceived as unexpected and shocking. One woman explained that she thought her baby would definitely die, and another woman described it as ‘feeling the world crumbling under my feet’ (participant #8). When the suspected diagnosis of SGA was confirmed in the hospital, the care was transferred from midwifery to the hospital in order to closely monitor the fetal condition by frequent ultrasounds.

### Theme 1: Experience of the ultrasound

#### Ultrasound as a necessary good

Having to come to the hospital on a frequent basis was time consuming for all women, and logistical factors such as long waiting times and scheduling issues were mentioned during most interviews. Nonetheless, women explicitly pointed out that that did not matter as long it was in the interest of the baby. Several women pointed out that the ultrasound was a necessary good. Also, getting the opportunity to see their baby was a special moment, ‘like a present’ and ‘giving energy’. This is the reason that most looked forward to the ultrasounds to ‘see the baby and check whether all is well’. All women, except for two, described anxiety building up prior to each ultrasound, but feeling reassured and relieved afterwards.

“Before the ultrasound? Tension. It’s like you have a checklist and each time when you walk out [the hospital] you tick a box: we are one week further, one day further. And approaching the next ultrasound a week later, the tension rises again. Yes, you live off the results of the ultrasounds.”

(participant #7, 27 weeks pregnant)

Negative experiences with the ultrasound itself were rare, although two women feared that the ultrasound could harm or hurt their baby.

#### It is not about my preference, it is about the baby growing

With regards to the ultrasound frequency, all women but one expressed no specific preferences and were fine with how often the ultrasound was repeated at that moment. The frequent ultrasounds felt ‘safe, that things are under control’ and provided comfort. Two women specifically reasoned to leave it up to the doctor, because they assumed the doctors would let them come only if it was necessary. Three women would have ideally seen their baby daily by ultrasound, but also believed that that would not be realistic for several reasons: logistical issues, fear that the baby would not like to be ‘poked in the side every day’ (participant #6), and simply because it was not about their preference, but about the baby growing every week. Only one woman wondered whether the weekly Doppler measurements were necessary, and would not have minded to only come fortnightly for the growth measurement. Nonetheless, she did mention to feel like the doctor knew better than her.

#### Silence is frightening

All women emphasized how much it was appreciated when the ultrasonographist continuously explained what was visible on the screen, and what was being measured and why:

“After all, they are going to measure a blood vessel in the baby’s brain, so of course I would like to know why.”

(participant #9, 26 weeks pregnant)

Silence was often interpreted by women as an indication that something might be wrong with their baby. Women then became attentive to the ultrasonographist’s facial expression and reaction to measurements. Silence was perceived as ‘frightening’ and a continuous explanation was preferred, including an immediate indication of measurement result. This provided confirmation and a feeling of security for the participants. Also, it was much appreciated when the ultrasonographist took the time and deviated from the medical focus by, for example, showing the face of the baby and printing a picture. One woman explained her definition of a good ultrasound.

“…when someone explains what they see during the ultrasound, because I don’t understand everything that I see. So, when someone just continuously talks and explains what is seen. Not just ‘this is the head’ or ‘the belly’, but also like ‘the bladder is nicely filled’. Using positive or negative words is something I really appreciated, so you know right away.”

(participant #6, 25 weeks pregnant)

Some women experienced differences in explanatory language between different ultrasonographists: ‘it depends on the person, and that is what I have realized’ (participant #1). Some employed more medical terminology, and others more easy terminology. Where some women preferred the first (‘we have perfect capability of understanding’), others preferred the latter, as they were ‘unfamiliar with the medical jargon’. Still, whatever terminology was used, adequate explanation of the purpose and findings during the ultrasound was much appreciated.

#### Ultrasound content

With regards to the ultrasound content, most women seemed well informed and some could even recount all measurements that were performed from the top of their head:

“It is basically the head, the femur, the circumference of the belly, and then you got two Dopplers, which are to the brain and to the umbilical cord to see the resistance of the flow.”

(participant #1, 31 weeks pregnant)

Women described different reasons for this high level of understanding: explanation of the ultrasonographist (‘sometimes I have an ultrasonographist that explains very well’), experience after having had multiple ultrasounds (‘I now know where to look at on the [ultrasound machine’s] screen’) and, in some cases, after having consulted the internet (‘I have done my own research’). The growth measurements were most easy to follow during the ultrasound for most women (‘I cannot see the values of the Doppler measurements that quickly, but for growth I know where to look [during the ultrasound]’). However, women had different opinions as to which measurement was most important to them. One woman explained:

“That [growth measurement] is not the only thing of course. Growth is a characteristic of the placenta working less well, but I think it is more serious when you suddenly see that the resistance in the umbilical cord is interrupted every time. You know, of course, these are all things that can tell if it is going bad, next to just growth. But the growth is still important, because [the baby] is very small and lagging behind.”

(participant #13, 30 weeks pregnant)

### Theme 2: Uncertainty and how to cope with it

Most women recognised the great uncertainties that come with the diagnosis of SGA regarding the causes and prognosis. They reported this as one of the biggest difficulties.

“Despite them [medical doctors] explaining everything that could happen, it remains an ungraspable phenomenon: it could be really bad, but it could just as well be nothing. […] Her growth determines whether you get scenario 1 or 30. […] Not knowing what to think of this, I find that difficult.”

(participant #2, 34 weeks pregnant)

Different ways of coping with these uncertainties were described. Several women accepted and recognised the uncertainties regarding the prognosis–‘nobody knows’ and ‘it’s just the way it is’. Some also explained that they did not expect their caregivers to be able to change this and had trust in the care they received, which enabled them to cope with the uncertainty. Contrastingly, the uncertainties surrounding SGA and the fact that it cannot be treated, left some women feeling powerless and like being in an emotional ‘rollercoaster’. One woman described:

“It is sort of like they are trying to scare me and I am trying to relax and not get stressed in the last few months of my pregnancy. […] So it is not that I am nervous about the ultrasounds, it is just that I don’t trust them [the doctors] to be very thorough and give me enough time to make a decision. […] I just feel like I am not being listened to and I am treated like they are the authority and I don’t know anything.”

(participant #12, 32 weeks pregnant)

Rationalising was another method of coping. All women looked for an explanation of SGA after their diagnosis, for example from previous pregnancies or from the fact that both parents were small in height. All of the seven multiparous women recounted that their other children had a low birth weight as well. Some even speculated that these children too might have been growth restricted and that this had been missed. This provided comfort to these women and helped them to rationalise that their current pregnancy would likely have a similar positive outcome as the previous one(s) did. Many nulliparous women indicated that both themselves and their partner were constitutionally small and had been as babies. They believed that as a result their baby was also constitutionally small and that, therefore, they felt that the SGA diagnosis was probably nothing to worry about. The use of Dutch growth charts was a specific point of discussion for foreign women, because ‘Dutch people are the tallest people in the world, so you are really comparing to something’ (participant #12). According to the women, this kind of reasoning was also frequently used by the healthcare providers in order to provide comfort.

“In India, we are very short and our babies are very small. The gynaecologist told me that the baby could be small because we are small.”

(participant #5, 26 weeks pregnant)

Conversely, one woman had over-rationalised by blaming herself for having exercised too intensively and having eaten too much fast food. Another coping mechanism used was comforting and wishful thinking. Several women reported to feel relieved when a certain gestational age was passed, so that the baby would not be born extremely preterm. Most women focused strongly on the growth of their fetus and gained hope and strength, when the growth was ‘in its own line’ or sometimes even ascended a few percentiles.

“It is inside the curve, which is what I was expecting, hopefully she gets a little bit … more, maybe she can reach to ten or something.”

(participant #1, 31 weeks pregnant)

### Theme 3: Information need and support

#### Gaps in information

Many women expressed a need for more information and counselling about the diagnosis and the possible causes of SGA.

“At no point have they said ‘yes you have this growth restriction’, because I didn’t get any information on that, they just kept saying the baby is small”

(participant #12, 32 weeks pregnant).

One participant explained that she had to actively gather the needed information in order to gain a complete picture of: “the phases, the consequences, if and when I can return to the midwife-led care, and what would happen in case the SGA would worsen” (participant #9, 26 weeks pregnant). Multiple women expressed the need for a specific intake appointment with a medical doctor to explain SGA and everything they could expect following the diagnosis. Two women suggested developing a leaflet for this purpose. Contrastingly, one woman was satisfied with the counselling, emphasising that it was explained clearly to her that her baby was too small, because the placenta did not function well, and another woman specified the many different appointments actually had given her a good understanding of her fetus’ condition.

Also, it was unclear to many women at which gestational age the delivery would be induced and what they could expect from labor induction. One woman indicated that she was referred to a leaflet about delivery, which made her feel as though the medical doctor was not interested in answering her questions. Additionally, two women preferred to receive more information about having a premature baby and the neonatal department.

Besides counselling on medical aspects, multiple participants expressed a lack of counselling ‘for you as a mother-to-be’, regarding the non-medical, practical aspects of pregnancy and childbirth. Especially for nulliparous women this was an important aspect, as they had never experienced pregnancy before. For this reason, three of the 15 participants chose to continue seeing their midwife even though their care had been transferred to the hospital. Apparently, not all women were aware of the frequent information evenings on delivery and consultation hours with an obstetric nurse that were actually held in the hospital. One woman added that she did not feel comfortable asking these general or ‘simple’ questions to her obstetricians, as they already appeared ‘very busy and always running behind schedule’ (participant #3, 27 weeks pregnant), although the participants did mention the obstetrician always asked them about any additional questions. It therefore appears that some women experience a barrier in requesting the obstetrician for more information, an issue they do not encounter when speaking with their midwife. Contrastingly, one woman was positive about the information she had received regarding delivery, having had a separate appointment with a clinical midwife to discuss the delivery, whereas another woman indicated she was content with the leaflet and information evening about delivery.

#### Support from partner and others

In general, the interviewed women received most emotional support from their partner. One woman explained that her relationship with her partner had grown stronger because of what they had been through together. Family and friends were also considered important. However, one woman felt agitated having to explain the difficult situation to all her family and friends: “After having received ten phone calls on one day, then I really don’t feel like [explaining] anymore. We have quite a large network and everyone knows that we are pregnant” (participant #8, 23 weeks pregnant).

Participants were invited to bring a companion of their choice to the ultrasounds and subsequent appointments, and more than half of the participants always did so. Most brought their partner, but sometimes a relative or friend came along. The main reason for women to bring a companion was to provide emotional support. For several women the moment of SGA diagnosis had been quite a stressful event, therefore, they preferred to have their partner present at each new appointment in case a similar situation occurred. As the situation could change with each ultrasound, women also preferred to bring a companion to have an extra pair of eyes and ears, someone to remember facts, data and ask questions.

### Theme 4: Continuity of care and satisfaction

#### Seeing different medical doctors

Seeing many different medical doctors negatively influenced the perceived quality of care for most women. Some explained that different doctors could provide contradicting information regarding the severity of the SGA and the moment of labor induction. This caused feelings of doubt and insecurity in the women.

“[The recommended date for induction] starts to become something like a ‘point of view’, which is quite uncertain for us.”

(participant #1, 31 weeks pregnant)

Despite using an electronic patient record system, many women experienced differences between doctors in the extent of familiarity with their case. Having to repeat their story at each new appointment felt ‘exhausting’ and like a waste of time. Some reported feeling like no one took responsibility and ownership of their case, causing the necessity to ‘fact check all the time’. They understood that appointing one permanent doctor might not be feasible in a large academic hospital, they nevertheless believed two or three doctors would be. They felt that this would allow them to build a relationship with their caregiver, and that this would improve the quality of care due to an increased familiarity with their case. In contrast, one participant reported not minding to see different doctors, as this made her feel more widely informed.

#### Seeing different ultrasonographists

Seeing different ultrasonographists was not reported as such a big issue, since the ultrasonographists generally do not make recommendations regarding the care of the patient. However, receiving the results of the ultrasound was an important aspect requiring more coordination and consistency, according to the participants. Some ultrasonographists gave the results during or directly after the ultrasound, while others gave only a vague indication of the results or no result at all, as these would be given by the medical doctor in a separate appointment after the ultrasound. Two women recounted that their ultrasonographist had provided results that were later disputed by the doctor, which had resulted in confusion. Some participants preferred to receive the results directly from the ultrasonographist, as this allowed them to better prepare for the consultation with the doctor. Other participants preferred not to hear the results from the ultrasonographist, pointing out that an ultrasonographist is not a doctor and is, therefore, not qualified to interpret the measurements. They felt that only the doctor would be able to assess the total case and interpret the results correctly.

Another interesting issue raised by the women, is that they felt that seeing many different ultrasonographists could negatively affect the accuracy of the measurements. They questioned the comparability of the measurements, when performed by a different individual on each occasion. Conversely, one woman considered seeing different ultrasonographists as an advantage, because ‘more people see more, you kind of get a second opinion each time’ (participant #14, 29 weeks pregnant).

## Discussion

### Main findings

This study explored experiences of monitoring the fetal condition by ultrasound in the outpatient clinic, among pregnant women diagnosed with an SGA fetus. Women in this study were content with the frequency of ultrasounds for their SGA pregnancies, and the frequent ultrasounds provided comfort and a feeling of safety. It was considered necessary, in the best interest of the baby, which outweighed the discomfort caused by having to come to the hospital frequently. Women described anxiety building up prior to each ultrasound, but feeling reassured and relieved afterwards. During the ultrasound a continuous explanation was preferred, which provided confirmation and a feeling of security. Nevertheless, many women had difficulty coping with the uncertainties regarding causes and prognosis after receiving the diagnosis of SGA. Women identified several areas for improvement to help coping with these uncertainties: providing better counselling and support regarding both medical and non-medical aspects of pregnancy and delivery, and safeguarding continuity of care.

### Strengths and limitations

As far as we know, this study is the first and only qualitative study addressing pregnant women’s experiences and preferences who are monitored for SGA. It adds to the large existing body of quantitative research on SGA[1] by providing a new, previously unexplored perspective: that of the pregnant woman. This article specifically addressed women’s experiences in The Netherlands. Differences between countries will exist, but many similarities are evident and the findings of this study can therefore be cautiously translated to most Western countries. A possible limitation is that the study was conducted among women attending one academic hospital that generally covers a higher socio-economic population of a large city. Although we tried to recruit a varied sample, women with a low socio-economic status were not included. This limits the generalizability of this study. However, the sample was varied as regarding medium and high socio-economic status, as regarding country of origin and as regarding timing of the interview after the diagnosis. Selection bias has possibly occurred: women may have been more inclined to participate when having criticism they wanted to share, while experiencing too much stress may have been less inclined to participate. The credibility of the process and of the results is enhanced by the analysis being undertaken by a multidisciplinary team to ensure that themes were robust and agreement was reached. Another strength is that this study adhered to the COREQ checklist for reporting of qualitative studies.[9]

### Interpretation

The need for greater understanding and acknowledgment of the patient’s experience is becoming increasingly prioritized by caregivers.[12] Our objective was to investigate women’s experiences of monitoring their SGA pregnancies. In terms of the ultrasounds, women experienced the frequent ultrasounds positively, providing comfort and a feeling of safety. It was considered necessary, in the best interest of the baby, which outweighed the discomfort caused by having to come to the hospital frequently. This is consistent with the results of a recent study, which assessed women’s experience of wearing a portable fetal-electrocardiogram device in their home environment to monitor SGA fetus.[13]

Nonetheless, women experienced difficulties coping with the uncertainties that come with the diagnosis of SGA regarding its prognosis. These uncertainties are inherent to SGA, since it is difficult to predict which fetuses are at risk and which are in fact just physiologically small.[5] According to the transactional theory, coping involves “constantly changing cognitive and behavioural efforts to manage external and/or internal demands that are appraised as taxing or exceeding the resources of a person”.[14] Coping strategies aim to either directly manage the stressor (problem-focused coping) or regulate emotions arising as a consequence of the stressful encounter (emotion-focused coping).[14] As far as we know, no studies exist on coping strategies in pregnancies complicated by SGA. Once SGA is diagnosed, there is little women can do themselves to improve the fetal growth. Consequently, participants in this study seemed to mainly describe emotion-focused coping strategies. This included rationalising of SGA by comparing the smaller weight of the fetus with their own height and country of origin, and with the birth weight of previous children. Other coping strategies described by the women were acceptance and trust, comforting and wishful thinking, and looking for information and support. Avoidant coping styles and behaviors were here generally not employed, whereas these are known to be associated with postpartum depression, preterm birth and infant development.[15]

However, women in this study described several gaps in the provision of information and support that deserve to be addressed in order to reduce the pregnant woman’s stress. To some extent, this feeling was likely amplified by their high educational status and the unsure implications of SGA and the future prognosis for their baby. As a comparison, two other studies on women’s perspectives on decision-making when a fetal malformation was detected by ultrasound, described that the unsure implication of the fetal anomaly was the most common reason for a feeling of inadequate information.[16, 17] Still, the experienced gaps could be largely closed by improving both healthcare coordination and patient-healthcare provider interaction in order to reduce the pregnant women’s stress. Healthcare coordination could be improved by offering a separate appointment with a specialized obstetrician, and by better continuity of caregiver. Most women thought that this would allow them to build a relationship with a caregiver, who also took responsibility for their case. The patient-healthcare provider interaction could be further improved, by continuous explanation during the ultrasound, but also by not rushing appointments, listening to the patient, and providing clear information. At the beginning of the appointment, the healthcare provider could ask the patient’s preference and needs for information, and ask for feedback and read back at the end of the appointment. All these items should be addressed in the training of medical doctors and ultrasonographists.

Interestingly, some women feared that seeing different ultrasonographists could negatively affect the accuracy of measurements. It is known that inter- and intraobserver variability of ultrasonic biometry measurements can indeed be large[18], and caregivers consider potential variability in measurement between ultrasonographists, when they interpret fetal growth rates. Explicitly explaining this to women appears to be important.

Continuity in a broader sense seemed to be important to women as well and could be improved, with a team of midwives and medical doctors sharing the same information and advice during all episodes of care, substantiated by consistency regarding guidelines. The extent to which continuity of care matters to pregnant women has been extensively discussed, and both caseload midwifery models (where women are allocated a primary ‘known’ midwife) [19–22] and team midwifery models (that include four to 12 midwives) [23] have been shown to increase women’s satisfaction. In the context of SGA, it appears that increased satisfaction can be primarily achieved by a team model, that safeguards continuity of information and care. A caseload model would be difficult to implement in the clinical practice of a large, academic hospital. Besides, a review by Green et al.[24] suggested that during labour individual caregivers’ approaches to care rather than a known caregiver lead to increased satisfaction. Continuity of information and care could be improved by targeted education for the medical doctors about the SGA care pathway. Such a care pathway could even be handed to the woman in the form of a leaflet to provide guidance throughout their complicated pregnancy.

Several opportunities for future research exist. It would be interesting to repeat this study in a more diverse study population, that also includes a substantial number of participants with low socio-economic status and education. The role and perspective of the woman’s partner could then also be further explored. By organising focus-groups, patient’s preferences towards the role and content of text-based counselling materials could be further explored. Lastly, we believe that patient input is of great importance for future quantitative studies on SGA. Women who have experienced an SGA pregnancy should be involved in discussing areas for further studies that aim to optimize monitoring and management of SGA. Such studies could lead to improved consensus between doctors and continuity of care and, consequently, to improved perceived quality of care.

## Conclusions

In the context of ultrasonographic monitoring of SGA pregnancies, women in this study were satisfied with the frequency of their ultrasounds, which provided a feeling of safety, despite experiencing a feeling of uncertainty regarding the causes and prognosis. From their ultrasonographists they sought continuous reassurance during the ultrasound. From their medical doctors they sought adequate counselling regarding both medical and non-medical aspects of their pregnancy complicated by SGA, which should be provided to them in a consistent and continuous manner by their medical team. Caregivers need to appreciate women’s uncertainty after SGA diagnosis and need to ensure they support and manage women’s expectations.

## Supporting information

S1 Appendix Interview guide. (DOCX)
