# Supplementary material for: Women’s experiences of monitoring the small-for-gestational age fetus by ultrasound: A qualitative study
Source: PLoS One. 2019 May 1;14(5):e0216052. doi: 10.1371/journal.pone.0216052 (PMC6493740; doi:10.1371/journal.pone.0216052)
Supplement: S1 Appendix — (DOCX) [file pone.0216052.s001.docx]

**S1 Appendix: Interview guide**

**A. Version in English:**

Researcher introduces herself.

Short explanation of the purpose of the interview.

Start the interview (start recording).

**INTRODUCTORY QUESTIONS**

- Can you tell me something about yourself? How many weeks are you pregnant? How are you doing?

**OPEN PHASE**

- Could you explain about when and how you heard the baby was not growing well?
- What are your expectations of the ultrasound monitoring?
- How do you experience the ultrasounds?
- Are you satisfied with the ultrasounds? Why, why not?
- Information regarding the ultrasound:
  - Do you know what the purpose is of the ultrasound?
  - How do/did you receive the results?
  - What do you feel/think about this?
  - Are/were the measurements done during the ultrasound understandable for you? Why, why not?
  - Which information is/was most important to you?
- How do you feel you are treated at the hospital visits?
- How often would you prefer the ultrasounds to be done? Why?
- How would you prefer to receive the results of the ultrasound? Why?
- Is there something you would like to change about the ultrasound monitoring? (Why (not)?
- Would you like to add anything?

**FOLLOW-UP QUESTIONS**

- Could you tell me more about that? Can you give an example?
- Could you tell me what your thoughts were then?
- What does that mean to you, personally?
- Did you doubt about what to do? What were your doubts about?

Researcher thanks participant and ends the interview.

--------------------------------------------------------------------------------------------------------------------------------------

**B. Version in the original language (Dutch):**

Voorstellen van onderzoeker.

Beknopte uitleg over het onderzoek en het interview.

Dan wil ik nu graag beginnen met het interview. Start geluidsopname.

**INTRODUCTIEVRAGEN**

- Kunt u uw kort iets over uzelf vertellen? woonsituatie of gezinssamenstelling kort omschrijven? U bent: getrouwd/samenwonend/alleenstaand/al andere kinderen?
- Hoeveel weken bent u zwanger? Hoe gaat het?

**OPEN FASE**

Ik wil graag met u teruggaan naar het moment in uw zwangerschap dat u hoorde dat uw ongeboren baby intra-uteriene groeirestrictie heeft. Kunt u vertellen hoe dat ging?

- Had u voordat u het wist, al eens gehoord van intra-uteriene groeirestrictie (verminderde groei van de baby)?
- Wat zijn eerdere ervaringen met echo’s?
- Wat zijn uw verwachtingen van de echo’s? Weet u waarom u de echo’s krijgt?

Ervaring echo:

- - Kunt u beschrijven hoe u zich voelt na de laatste echo, ten opzichte van ervoor?
  - Hoe voelt u zich voor de echo?
  - Kunt u beschrijven hoe de echo verliep? Alleen of met partner/familielid?
  - Hoe voelde u zich tijdens de echo, wat vond u wel of niet prettig?
  - Hoe voelde u zich na de echo?
  - Hoe ervaart uw partner de echo’s?

Informatie echo

- Hoe ontvangt u de uitslag van de echo’s en wat vind u hiervan?
- Van wie en op welk moment heeft u de uitslag ontvangen? Hoe vond u dat?
- Wat was de uitslag? Wat betekent dit voor u?
- Wat vind u van de manier waarop u de uitslag te horen kreeg?

Wat vond u van de begrijpelijkheid van de informatie over:

- - Welke metingen worden er gedaan tijdens de echo? Wat wordt er precies gemeten?
  - Hoe begrijpelijk vind u de metingen?

Welke informatie (vorm, inhoudelijk) was voor u meest waardevol?

- Welke meting vindt u het *meest* belangrijk tijdens de echo?
- Welke meting vindt u het *minst* belangrijk tijdens de echo?

Extra: Hoe heeft u de counseling en ondersteuning (los van de informatievoorziening) ervaren?

Heeft u het gevoel dat u voldoende kan meebeslissen in uw zorg?

Wordt er voldoende rekening gehouden met uw wensen en voorkeuren?

Krijgt u voldoende de kans om uw zorgen te bespreken?

Voorkeur

- Hoe kijkt u terug op de echo? Bent u tevreden met de echo? Zo ja, waarom? Zo nee, waarom niet?
- Met wie gesproken? Vind u dat u goed behandeld wordt?
- Hoe vaak zou u het liefst een echo willen? Heeft u nog zorgen over het aantal echo’s?
- Wat vind u van de duur van de echo? Wilt u graag meekijken tijdens de echo?
- Hoe zou u het liefst de uitslag van de echo horen?
- Wat vind u van de bewaking van uw privacy?

Nog een laatste vraag: Als u 1 ding zou kunnen veranderen aan de (informatie) die u over echo hebt gekregen of hoe de uitslag werd doorgegeven naderhand, wat zou u dan veranderen?

**STANDAARD FOLLOW-UP VRAGEN**

- Waarom vond u dat?
- Kunt u daar meer over vertellen?
- Kunt u terughalen wat u toen dacht of heeft ervaren?
- Wat betekent dat voor u persoonlijk?
- Heeft u toen getwijfeld over wat u zou doen? Waar had u twijfels over?

Bedanken en afsluiten

--------------------------------------------------------------------------------------------------------------------------------------

**C. Questionnaire for participant’s background information**

| **1.** | Wat is de datum waarop u deze vragenlijst invult? | \|___\|__5_\| dag \|_1__\|_2__\| maand \| 2 \| 0 \| \|__1_\|__7_\| jaar | |
| --- | --- | --- | --- |
| **2.** | Hoe lang geleden heeft u uw laatste echo gehad? | \|_0__\|___\| dag \|_1__\|___\| uur | |
| **3.** | Hoeveel weken bent u momenteel zwanger? | _29__ weken | |
| **4.** | Wat is uw leeftijd? | __32_ jaar | |
| **5.** | Is dit uw eerste zwangerschap? | □ Ja  □ Nee, namelijk de ____ zwangerschap (aantal invullen) | |
| **6.** | Hoeveel kinderen heeft u op dit moment? | __1__kinderen | |
| **7.** | Hoe zou u over het algemeen uw gezondheid noemen? | □ Uitstekend  □ Zeer goed  □ Goed  □ Matig  □ Slecht | |
| **8.** | Wat is uw hoogst afgemaakte opleiding?  (één antwoord aankruisen) | □ Geen, lagere school  □ LBO, MAVO  □ VMBO  □ MBO, HAVO, VWO  □ HBO, Universiteit  □ Anders, namelijk…………………………………………………………. | |
| **9.** | Wat is uw burgerlijke status? | □ Alleenstaand  □ Samenwonend/gehuwd  □ Anders, namelijk…………………………………………………………. | |
| **10.** | In welk land bent u geboren? | NL………………………………………………………………………………………… | |
| **11.** | In welk land is uw vader geboren? | ……NL…………………………………………………………………………………… | |
| **12.** | In welk land is uw moeder geboren? | ……NL…………………………………………………………………………………… | |
| **13.** | Tot welke bevolkingsgroep rekent u zichzelf? | □ Nederlands  □ Turks  □ Marokkaans | □ Surinaams  □ Antilliaans  □ Anders, namelijk………… |
| **14.** | Tot welke kerkelijke gezindte of levensbeschouwelijke  groepering rekent u zichzelf? | □ Geen  □ Rooms-katholiek  □ Nederlands Hervormd  □ Gereformeerde kerken  □ Protestantse Kerk | □ Islam  □ Joods  □ Hindoe  □ Boeddhist  □ Anders, namelijk………… |
| **15.** | Hoe actief bent u in uw geloof? | □ Erg actief  □ Enigszins actief | □ Niet actief  □ Niet van toepassing |
